# A United Approach to Enhance Adolescent Health Measurement

**DOI:** 10.1016/j.jadohealth.2023.12.002

**Published:** 2024-06

**Authors:** Parviz Abduvahobov, Regina Guthold, Ann-Beth Moller, Howard S. Friedman, Liliana Carvajal-Velez, Victoria Bendaud, Nazneen Damji, Andrew D. Marsh, Christopher Castle, Anshu Banerjee

**Affiliations:** a Division for Peace and Sustainable Development, Health and Education Section, Education Sector, UNESCO, Paris, France; b Maternal, Newborn, Child and Adolescent Health and Ageing Department, WHO, Geneva, Switzerland; c UNDP/UNFPA/UNICEF/WHO/World Bank Special Programme of Research, Development and Research Training in Human Reproduction (HRP), Department of Sexual and Reproductive Health and Research, World Health Organization, Geneva, Switzerland; d Technical Division, United Nations Population Fund, New York, New York; e Division of Data, Analytics, Planning and Monitoring, Data and Analytics Section, UNICEF, New York, New York; f Data for Impact Practice, UNAIDS, Geneva, Switzerland; g Governance and Participation Section, Gender equality, HIV and Health, UN Women, New York, New York; h Division for Peace and Sustainable Development, Education Sector, UNESCO, Paris, France

Adolescence has been recognized as a pivotal period for individuals to acquire the critical physical, cognitive, emotional, social, and economic resources and skills underpinning lifelong health and well-being [1]. Consequently, the need for high-quality data on adolescent health has gained attention in the global development agenda, as demonstrated in the Sustainable Development Goals, the Global Strategy for Women's, Children's and Adolescents' Health, and the Global Accelerated Action for the Health of Adolescents, among others[[2], [3], [4]]. The United Nations (UN) partners have acknowledged the imperative of meeting this demand while also addressing key data-related challenges, such as avoiding duplication, reducing country reporting burden, leveraging existing mechanisms, and achieving greater harmonization, as outlined in various international calls for action, including the Paris Declaration on Aid Effectiveness and the Accra Agenda for Action [5].

The Global Action for the Measurement of Adolescent Health (GAMA) Advisory Group (AG) was established in 2018 by the World Health Organization (WHO) in partnership with the Joint UN Programme on HIV/AIDS, UN Educational, Scientific and Cultural Organization, UN Population Fund, UN Children's Fund, UN Women, the World Bank Group, and the World Food Programme in response to this particular challenge [6]. The GAMA AG, composed of 16 global experts in adolescent health measurement from 12 countries across various geographic regions and income levels, has set itself to consider some of the perennial challenges facing the field of adolescent health measurement (Panel 1).

Panel 1 **Questions Considered by the Global Action for Measurement of Adolescent Health Advisory Group**

1. How can we bridge the knowledge and accountability gap, considering the varying quality and scarcity of data required for evidence-based planning, investment, policy-making, and programming to enhance adolescent health? [9].
2. How can we establish consistency in measuring adolescent health across and within countries, ensuring comparability of data despite variations in contextual priorities and measurement approaches? [8].
3. How can we achieve a balanced approach to addressing key adolescent health topics, ensuring that both topics receiving significant attention and those neglected are appropriately accounted for, with relevant indicators and appropriate data sources proposed for measurement and monitoring? [12].
4. How can we develop a globally relevant indicator set encompassing well-established indicators for which data are readily available as well as critical indicators with limited or no data availability, while minimizing the data collection burden on countries?

GAMA has embarked on an ambitious and bold approach to define a set of priority indicators for adolescent health measurement. Led by the Maternal, Newborn, Child, and Adolescent Health and Ageing Department and supported by 11 other relevant departments, WHO plays a crucial role by providing a Secretariat function to the GAMA AG. Furthermore, a Steering Committee comprising UN agency representatives was formed to strategically respond to the recommendations of the AG, facilitate effective communication between AG and respective UN agencies, and ensure that insights from the AG inform UN guidance and programmes.

Since its inception, GAMA has achieved several significant milestones. These include identifying priority areas for measuring adolescent health based on disease burden, existing initiatives, inputs from countries and youth representatives [7], mapping 413 existing adolescent health indicators [8], and selecting a draft list of 52 priority (36 core, one alternative, and 15 additional) indicators [9]. As work continues, the focus remains on essential tasks leading up to the launch of the indicators. These include refining the set of priority indicators based on the 12-country feasibility study, harmonization exercise and data availability assessments for the indicators, developing guidance and technical support materials for the implementation at the country level, and ensuring widespread dissemination of measurement guidance to facilitate effective utilization by stakeholders and countries.

Following the launch of the indicators, three foreseeable change pathways are envisioned, as outlined in the Theory of Change (Figure 1). Firstly, the priority indicators will inform global measurement initiatives and their underlying instruments, such as global household surveys, global school-based surveys, and cross-national learning assessments. This will prompt the inclusion or alignment of the indicators within these instruments, including in terms of disaggregation by the population of interest (adolescents in the age groups of 10–14 and 15–19), construct and behavior, threshold determination, and reference or recall period, where relevant. The evidence of this alignment is already apparent. GAMA has provided advice to the Measurement of Mental Health among Adolescents and Young People initiative [10] led by the UNICEF, resulting in the inclusion of key indicators on adolescent and young people's mental health as a complementary module in the seventh round of the Multiple Indicator Cluster Surveys [11].

**Figure 1:**
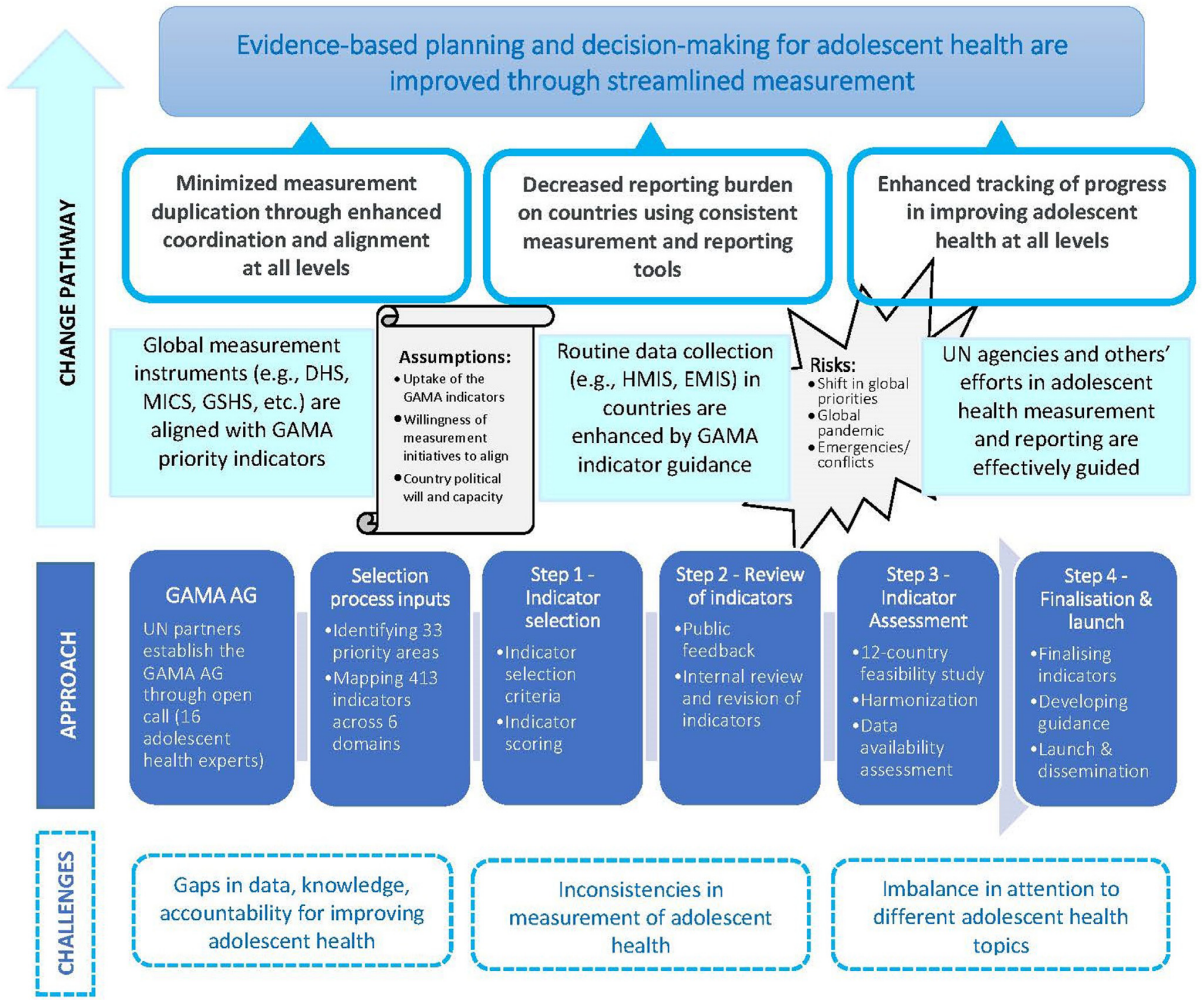
Theory of Change for the Global Action for the Measurement of Adolescent Health. Note: GAMA AG–Global Action for Measurement of Adolescent Health Advisory Group; DHS–Demographic and Health Survey; MICS–Multiple Indicator Cluster Survey; GSHS–Global school-based Student Health Survey; HMIS–Health Management Information System; EMIS–Education Management Information System.

Secondly, GAMA anticipates that the accompanying guidance and support tools for the indicators will contribute to improving the routine country-led collection of age-disaggregated and sex-disaggregated data on adolescent health. In pursuit of this, particular emphasis will be placed on utilizing existing systems, such as health and education information management systems, to avoid duplication of measurement efforts while fostering greater ownership, evidence-based planning, and decision-making. Thirdly, we expect GAMA will persist in effectively guiding the policy and programming of UN partners in their adolescent health measurement efforts.

Like any collaborative effort, this approach has strengths and limitations that must be acknowledged. One challenge is the complexity of coordinating multiple stakeholders' involvement with varying priorities. Another is reflecting different country contexts. Moreover, varying patterns of rapidly evolving and emerging health needs [6], particularly in the post–COVID-19 period, add to the complexity and necessitate a periodic review of the indicators to ensure their relevance, not only in development contexts but also in crises and emergencies. However, it is essential to highlight that the strengths of this approach outweigh the constraints. The selection of priority indicators underwent a rigorous process, including wide-ranging consultations and field testing, ensuring their relevance, feasibility, and usefulness. By working together, the UN agencies can leverage their collective expertise and resources, speaking with a unified voice to drive progress in adolescent health measurement and ultimately strengthen global efforts in improving adolescent health outcomes. GAMA's approach is an excellent example of the harmonized and collaborative efforts of UN agencies and provides a promising model for future endeavors.

## References

[1] Patton G.C., Sawyer S.M., Santelli J.S. (2016). Our future: A Lancet commission on adolescent health and wellbeing. Lancet.

[2] United Nations (2017).

[3] Every Woman Every Child (2015).

[4] World Health Organization (2017).

[5] OECD/DAC (2005/2008).

[6] Guthold R., Moller A.B., Azzopardi P. (2019). The global action for measurement of adolescent health (GAMA) initiative-rethinking adolescent Metrics. J Adolesc Health.

[7] Guthold R., Moller A.B., Adebayo E. (2021). Priority areas for adolescent health measurement. J Adolesc Health.

[8] Newby H., Marsh A.D., Moller A.B. (2021). A Scoping review of adolescent health indicators. J Adolesc Health.

[9] Marsh A.D., Moller A.B., Saewyc E. (2022). Priority indicators for adolescent health measurement - recommendations from the global action for measurement of adolescent health (GAMA) Advisory group. J Adolesc Health.

[10] UNICEF (2023). Measuring mental health for adolescents and young people at the population level. https://data.unicef.org/topic/child-health/mental-health/mmap/.

[11] UNICEF Multiple indicator Cluster surveys (MICS). https://mics.unicef.org/surveys.

[12] Adamou B. (2020).

